# Genomic selection shows improved expected genetic gain over phenotypic selection of agronomic traits in allotetraploid white clover

**DOI:** 10.1007/s00122-025-04819-w

**Published:** 2025-01-23

**Authors:** O. Grace Ehoche, Sai Krishna Arojju, M. Z. Zulfi Jahufer, Ruy Jauregui, Anna C. Larking, Greig Cousins, Jennifer A. Tate, Peter J. Lockhart, Andrew G. Griffiths

**Affiliations:** 1 Grasslands Research Centre, AgResearch Ltd, Private Bag 11008, Palmerston North, 4442 New Zealand; 2 Massey University, Private Bag 11222, Palmerston North, 4442 New Zealand; 3 Present Address: PGG-Wrightson Seeds , AgResearch Grasslands Research Centre, Palmerston North, New Zealand; 4 Present Address: Radiata Pine Breeding Company, University of Canterbury, Building EN27, Christchurch, 8041 New Zealand; 5 Present Address: Animal Health Lab, Ministry for Primary Industries, Wallaceville, New Zealand; 6 Present Address: School of Agriculture and Sustainable Food, The University of Queensland, Brisbane, QLD 4072 Australia

## Abstract

**Key message:**

Genomic selection using white clover multi-year-multi-site data showed predicted genetic gains through integrating among-half-sibling-family phenotypic selection and within-family genomic selection were up to 89% greater than half-sibling-family phenotypic selection alone.

**Abstract:**

Genomic selection, an effective breeding tool used widely in plants and animals for improving low-heritability traits, has only recently been applied to forages. We explored the feasibility of implementing genomic selection in white clover (*Trifolium repens* L.), a key forage legume which has shown limited genetic improvement in dry matter yield (DMY) and persistence traits. We used data from a training population comprising 200 half-sibling (HS) families evaluated in a cattle-grazed field trial across three years and two locations. Combining phenotype and genotyping-by-sequencing (GBS) data, we assessed different two-stage genomic prediction models, including KGD-GBLUP developed for low-depth GBS data, on DMY, growth score, leaf size and stolon traits. Predictive abilities were similar among the models, ranging from −0.17 to 0.44 across traits, and remained stable for most traits when reducing model input to 100–120 HS families and 5500 markers, suggesting genomic selection is viable with fewer resources. Incorporating a correlated trait with a primary trait in multi-trait prediction models increased predictive ability by 28–124%. Deterministic modelling showed integrating among-HS-family phenotypic selection and within-family genomic selection at different selection pressures estimated up to 89% DMY genetic gain compared to phenotypic selection alone, despite a modest predictive ability of 0.3. This study demonstrates the potential benefits of combining genomic and phenotypic selection to boost genetic gains in white clover. Using cost-effective GBS paired with a prediction model optimized for low read-depth data, the approach can achieve prediction accuracies comparable to traditional models, providing a viable path for implementing genomic selection in white clover.

**Supplementary Information:**

The online version contains supplementary material available at 10.1007/s00122-025-04819-w.

## Introduction

White clover (*Trifolium repens* L.) is an important legume grown in temperate regions of the world where it is valued for its quality forage and ability to provide plant-available nitrogen through symbiosis with soil-borne *Rhizobia* bacteria (Caradus et al. 1997; Jahufer et al. 2002; Weith et al. 2022). It is also a recent allotetraploid that has retained both its progenitor genomes, a feature which likely underpins the broad adaptation and phenotypic plasticity of this species and facilitates a global ecological niche expansion well beyond the restricted ranges of the progenitors (Griffiths et al. 2019). Its utility as a forage is demonstrated in numerous studies which have shown that cows grazing on grass/clover mixtures produce higher milk solid content and increased yields compared to those on grass monocultures (Dineen et al. 2018; Egan et al. 2018). While white clover is a common component in temperate livestock systems, its potential remains underutilized due to inconsistent vegetative persistence and seasonal yield. (Woodfield and Caradus 1994; Caradus et al. 1995). Improving these traits, both of which are quantitatively inherited, is a key focus in white clover breeding programmes; however, the historical realized genetic gain for dry matter yield (DMY) in white clover has been low, at only 0.16% per decade (Hoyos-Villegas et al. 2019). In addition, conventional forage breeding relies on evaluating candidates at the fully developed plant stage, which makes assessing DMY and persistence time-consuming and expensive. Breeding populations must be maintained over several years and locations to ensure broad adaptation, leading to inefficiencies as many individuals are discarded without being selected for desired traits. To improve efficiency, tools that allow for screening and identifying elite individuals at the seedling stage could greatly enhance genetic gains for DMY and persistence (Rutkoski et al. 2011; Bassi et al. 2016).

Genomic selection enables identification of selection candidates at the seedling stage by combining genome-wide marker profiles and multi-environmental trial (MET) phenotype information from a training population. This combination of datasets facilitates prediction of genomic estimated breeding values (GEBVs) in selection candidates based on marker information alone, without need for further METs (Jannink et al. 2010; Hayes et al. 2013). A major advantage of genomic selection is the reduction in time to complete a breeding cycle by selecting future candidates rapidly without the need for METs (Heffner et al. 2010; Resende et al. 2012). Furthermore, forage breeding often employs a half-sibling (HS) family strategy where only a quarter of the additive genetic variation is accessible among families, whereas three quarters is located within families (Falconer 1989). The lack of tools to access this within-family variation at high accuracy and low cost has been identified as a major reason for the poor genetic gains in forages (Casler 2008; Casler and Brummer 2008; Resende et al. 2014). Genomic selection can predict the GEBVs of individuals within the family, thereby accessing the three quarters of additive genetic variation from within the family and potentially accelerating genetic gains (Faville et al. 2018; Barre et al. 2022). However, the success of genomic selection relies on the ability to predict GEBVs at as high an accuracy as possible.

Accurate prediction of GEBVs is influenced by many factors, including trait architecture, genomic selection model, marker density, linkage disequilibrium (LD) and training population size (Zhong et al. 2009; Daetwyler et al. 2013; Isidro et al. 2015; Alemu et al. 2024). Assessing different prediction models and marker densities can influence predictive ability. Recently, multi-trait genomic selection has proved to be a promising approach to significantly boost GEBV accuracy by utilizing the genetic correlation from a secondary trait (Montesinos-López et al. 2019; Arojju et al. 2020b; Osterman et al. 2024). Multi-trait genomic selection was shown to be highly successful in scenarios where the primary trait of interest is of low heritability, such as DMY, and the secondary trait is a highly heritable trait with significant genetic correlation with the primary trait (Jia and Jannink 2012). Significant research has been focused on elucidating these contributing factors in major crop species and in forages such as perennial ryegrass (Fè et al. 2016; Faville et al. 2018; Arojju et al. 2020a; Barre et al. 2022), alfalfa (Annicchiarico 2015) and red clover (Skøt et al. 2024). While white clover is an important forage legume in temperate grasslands, there are few published studies that explore application of genomic selection for genetic improvement in this species. Moeskjær et al. (2022) reported positive prediction accuracies ranging from 0.16 to 0.53 for various growth traits derived from an image-based high-throughput phenotyping platform evaluated under glasshouse conditions. Additionally, Ehoche et al. (2021) reported a prediction accuracy of 0.48 for spring DMY based on 2-year field trial data using a training set of 200 HS families.

For many species, phenotyping costs have become a bottleneck in breeding, while genotyping costs are dropping steadily (Heslot et al. 2015). A case in point is white clover, which has a low rate of genetic gain mainly due to long breeding cycles, and expensive, difficult-to-measure traits such as those associated with vegetative persistence and DMY. For species with low-density genotyping resources, development of low-cost high-throughput genotyping platforms, such as reduced-representation genotyping-by-sequencing (GBS; Elshire et al 2011), has provided a route for implementation of genomic selection. However, a feature of GBS is the level of missing data due to the low sequencing depth which complicates generation of a genomic relationship matrix (GRM), a key requirement of two-stage genomic selection. A methodology, estimating kinship using GBS with depth adjustment (KGD), has been developed to generate GRMs from GBS data without imputation or heavy filtering to remove missing data (Dodds et al 2015). To our knowledge, comparison of the KGD method with other models in genomic prediction has not been performed in white clover. Furthermore, genomic selection feasibility studies on various agronomic traits based on field trial assessment and various factors influencing the predictive ability and accuracy have also not been reported in white clover. Our objectives were to use data from a multi-year, multi-site grazed field trial to: (i) investigate the feasibility of implementing genomic selection in white clover by ascertaining the influence of various factors such as population size, marker number, and prediction models on predictive ability, particularly when using a model (KGD-GBLUP) that does not require imputation for GBS data; (ii) assess the performance of single-trait and multi-trait genomic prediction models in terms of improving predictive ability; and (iii) compare and contrast the rate of predicted genetic gain based on among-family half-sib phenotypic selection (HS_P_) and a combined among-family phenotypic selection and within-family genomic selection (ApWFgs) breeding strategy.

## Materials and methods

### Training population, experimental design and evaluation of half-sib families

The breeding/phenotype population consisted of 200 F_3_ half-sibling (HS) families derived from broadly adapted white clover breeding lines. A detailed explanation of the training population development was described in Ehoche et al. (2022). The F_2_ parents of the HS families were genotyped and were the training population whose phenotypes were inferred from the performance of their corresponding F_3_ HS families.

As described by Ehoche et al. (2021) and Ehoche et al. (2022), the phenotyping population consisting of 200 F_3_ HS families were evaluated in a row-column design at two locations in New Zealand: AgResearch research farms Aorangi (Palmerston North, Manawatū (40.38˚S, 175.61˚E) and Ruakura ((Hamilton, Waikato (37.77˚S, 175.31˚E) with three replications per HS family. Location and meteorological characterization, trial establishment, companion perennial ryegrass details, cattle grazing, trial management and maintenance and trait measurement protocols were as described by Ehoche et al. (2022). In addition to the 200 F_3_ HS families, 24 repeated checks each of two cultivars ‘Grasslands Kopu II’ (large-leafed cultivar) and ‘Grasslands Bounty’ (small-medium leafed cultivar) were allocated within each of the three replicates to account for spatial trends across trials. This resulted in a total of 672 plots at each location and phenotyping was performed as described by Ehoche et al. (2022) for 3 years from August 2016 to November 2019. Traits measured at both locations were spring dry matter yield (DMY); seasonal growth scores (GS; 1 (lowest) to 9 (highest) herbage production per plot, with 0.5 unit increments to allow closer approximation of continuous data); and leaf size scores (LS; 1 (lowest) to 5 (highest), with 0.5 unit increments. Stolon density traits, namely pre-summer stolon number (SNPRS), pre-summer stolon branches (SBPRS), post-summer stolon number (SNPOS) and post-summer stolon branches (SBPOS), were measured at a single location (Aorangi).

### Statistical analysis

Phenotypic data were analysed using a univariate linear mixed model employing a residual maximum likelihood (REML) approach for all traits using DeltaGen (Jahufer and Luo 2018) software which is underpinned by the lme4 R (Bates et al. 2015) package. This analysis generated estimates of genetic and non-genetic variance components and best linear unbiased predictions (BLUPs) for all traits. A detailed assessment of various quantitative genetic parameters and linear mixed models employed were described in Ehoche et al. (2022). The principal model accounting for seasonal, year and location effects for estimating BLUPs to be used in genomic prediction modelling was Eq. 3 previously reported in Ehoche et al. (2022). The traits selected for genomic prediction modelling were, unless otherwise stated, spring DMY combined across sites and years; LS combined across sites and years; GS combined across sites and years; and stolon density traits pre- and post-summer (SNPRS, SBPRS, SNPOS, and SBPOS).

### Genotyping by sequencing and analysis

Genomic DNA was extracted from the 200 F_2_ maternal parents in a 96-well plate and quantification was performed as described in Anderson et al. (2018). Three 96-plex *Ape*KI genotyping-by-sequencing (GBS) libraries were constructed based on a method proposed by Elshire et al. (2011) with modifications as described in Faville et al. (2018). Sequencing was done on two lanes of Illumina HiSeq 2500 (Illumina, San Diego, CA, USA) at AgResearch Invermay, New Zealand. Single-end 150 nucleotide sequence reads were obtained for all three libraries, de-multiplexed and trimmed using Trimmomatic software (Bolger et al. 2014). A sliding window of 10% of the total read length was used to check quality, retaining only regions with an average q-score above 15. The TASSEL5 GBS pipeline was used to call genetic variants as described (Griffiths et al. 2019) by aligning to the *Trifolium repens* genome (version five; Griffiths et al. 2019) discovering a total of 361,220 single nucleotide polymorphisms (SNPs). After filtering for minor allele frequency (MAF) ≥ 0.01, missing rate > 50% and Hardy–Weinberg disequilibrium (HWdiseq > −0.05), the pipeline produced a variant call format (vcf) file with 110,000 SNPs with a median read depth < 12.8.

### Genomic prediction

A two-stage approach was implemented for both single and multi-trait genomic prediction. The first stage involved the estimation of BLUPs for each of the 200 F_3_ HS families and was performed using Eq. 3 reported in Ehoche et al. (2022). In the second stage, genotypic data generated from the 200 F_2_ maternal parents and the BLUPs estimated on the F_3_ progeny families for the traits with significant additive variation were fitted into single and multi-trait genomic prediction models.

### Single-trait genomic prediction

Four different genomic prediction models with different statistical assumptions were investigated for three different traits (spring DMY, GS and LS) in this study. Of these four models, only one (KGD-GBLUP; Dodds et al 2015) could use genotyping data with missing values and, therefore, did not require imputation. This methodology aligns well with the type of low sequence depth and corresponding high levels of missing data generated with the GBS platform. The most practical prediction model was determined via a Monte Carlo cross-validation approach based on the calculated predictive ability and bias. Predictive ability is the pairwise Pearson’s correlation coefficient (*r*) between the predicted values (GEBVs) and the observed values (BLUPs) in the test set, whereas bias is the regression coefficient of the observed phenotype data on the predicted value generated by the prediction model (Velazco et al. 2019b).

The first model, GBLUP, used a linear mixed model approach where the genomic relationship matrix (GRM) was included as a variance–covariance matrix in the model (Eq. 1). The relationship between individuals was estimated from the SNP markers under the assumption of equal variance across all loci (Habier et al. 2007). Missing values were imputed with the mean value of the non-missing values for that marker. The GRM was calculated using the ‘A.mat’ function from the ‘rrBLUP’ package (Endelman 2011) in R (R Development Core Team 2018) (R Development Core Team 2018) according to Eq. 2 as proposed by VanRaden (2008).

1 $$y = \mu + {\mathbf{Z}}b + \varepsilon$$

where *y* is the vector of phenotypic records; *µ* is the grand mean; ${\varvec{Z}}$ is the incidence matrix for random effects; and *b* is the vector of random marker effects and modelled as coming from a normal distribution with the given mean and variance N (0, ***G*** $\sigma_{g}^{2}$) where ***G*** is the GRM and $\sigma_{g}^{2}$ is additive genetic variance; $\varepsilon$ is the vector of random residual effects.

The GRM is calculated as follows:

2 $${\varvec{G}} = \frac{{{\varvec{XX}}^{\prime}}}{{2 \mathop \sum \nolimits_{i = 1}^{m} p_{i} \left( {1 - p_{i} } \right)}}$$

where ${\varvec{X}}$ is obtained by subtracting ***M*** *–* ***P***, ***M*** being a matrix with rows (*n*) and columns (*m*), containing markers coded as −1, 0, 1, and ***P*** is a matrix with MAF (minimum allele frequency) calculated as $2\left( {p_{i} - 0.5} \right)$ where $p_{i}$ is the MAF of the *i*th marker.

The second prediction model KGD-GBLUP is a variant of GBLUP, with the GRM calculated using the KGD method (Dodds et al. 2015). The KGD method was developed specifically to estimate relatedness from low-depth sequence data, which usually have high levels of missing data, without a need for imputation of SNPs. The GRM was estimated according to Eq. 2 with correction factors applied to account for low sequence depth and missing data using R scripts (Dodds et al. 2015).

The third model was BayesCπ, which assumes different degrees of variance for markers, thereby allowing markers to depart from normality rather than shrinking towards zero (Meuwissen 2003). Missing values were mean values imputed for the BayesCπ model. The prediction model was implemented using the ‘BGLR’ package (Pérez and de los Campos 2014) in R (R Development Core Team 2018) with the burn-in number set to 1500 and total number of iterations set to 3000.

The final model was Reproducing Kernel Hilbert Spaces (RKHS), a semi-parametric prediction model. In this model, a kernel function is employed to convert the marker matrix into distances between pairs of individuals forming a square matrix used in a mixed-effects linear model (Heslot et al. 2012). Missing values were mean values imputed for the RKHS model. The RKHS model was implemented using the ‘BGLR’ package in R (Pérez and de los Campos 2014).

The Monte Carlo cross-validation approach was used to assess predictive ability by splitting the data into training and test sets. The phenotypes of the individuals in the test set were assumed to be unknown and predicted from a model trained exclusively from the training set.

To investigate the effect of training population size and number of markers on predictive ability, GBLUP was selected as the model of choice considering the computational load and initial results. The influence of using fewer individuals in the training population was investigated by using subsets of 20, 40, 60, 80, 100, 120, 140, 160 and 180 HS families randomly chosen to train the model. The effect of marker number on predictive ability was also considered by selecting random subsets of markers (55, 110, 550, 1100, 5500, 11,000 and 55,000) for model development. For each population and marker subset, the process was repeated 100 times and the predictive ability represented as the average of these 100 iterations.

### Multi-trait genomic prediction

A multi-trait genomic prediction model was fitted using correlated secondary traits to boost the predictive abilities for primary traits. The primary traits used for multi-trait model development were spring DMY and stolon number (SN), whereas LS and GS were considered as secondary traits for spring DMY, and for SN, stolon branching (SB) was considered as secondary trait based on the genetic correlations. A multi-variate mixed model was fitted using two or more correlated traits as follows:

3 $$\left[ {\begin{array}{*{20}c} {\begin{array}{*{20}c} {y_{1} } \\ . \\ \end{array} } \\ . \\ {y_{o} } \\ \end{array} } \right] = \left[ {\begin{array}{*{20}c} {\begin{array}{*{20}c} {\mu_{1} } \\ . \\ . \\ \end{array} } \\ {\mu_{o} } \\ \end{array} } \right] + { }\left[ {\begin{array}{*{20}c} {{\varvec{Z}}_{1} } & 0 \\ {\begin{array}{*{20}c} . \\ . \\ \end{array} } & {\begin{array}{*{20}c} . \\ . \\ \end{array} } \\ 0 & {{\varvec{Z}}_{o} } \\ \end{array} } \right]{ }\left[ {\begin{array}{*{20}c} {\begin{array}{*{20}c} {b_{1} } \\ {\begin{array}{*{20}c} . \\ . \\ \end{array} } \\ \end{array} } \\ {b_{o} } \\ \end{array} } \right] + { }\left[ {\begin{array}{*{20}c} {\begin{array}{*{20}c} {\begin{array}{*{20}c} {\varepsilon_{1} } \\ . \\ \end{array} } \\ . \\ \end{array} } \\ {\varepsilon_{o} } \\ \end{array} } \right]$$

where $y_{1} \ldots y_{o}$ are the vector of BLUP values for $o$ traits; $\mu_{1} \ldots \mu_{o}$ are the vector grand means for $o$ traits; ${\varvec{Z}}_{1} , \ldots ,{\varvec{Z}}_{o}$ are the design matrices associated with random effects $b_{1} , \ldots ,b_{o}$, in which $$\left[ {\begin{array}{*{20}c} {\begin{array}{*{20}c} {b_{1} } \\ . \\ . \\ \end{array} } \\ {b_{o} } \\ \end{array} } \right] \sim N\left( {0, {\varvec{G}} \otimes {\varvec{K}}} \right)$$, where ${\varvec{G}}$ is the marker-based relationship matrix (KGD-GRM matrix) and $\user2{K }$ is the genetic variance–covariance matrix for $o$ traits and modelled as coming from a normal distribution with the given mean and variance; $\varepsilon_{1} , \ldots ,\varepsilon_{o}$ are the random residuals from bivariate models, with $$\left[ {\begin{array}{*{20}c} {\begin{array}{*{20}c} {\varepsilon_{1} } \\ . \\ . \\ \end{array} } \\ {\varepsilon_{o} } \\ \end{array} } \right] \sim N\left( {0, {\varvec{I}} \otimes {\varvec{R}}} \right)$$, where $\user2{I }$ is the identity matrix and ${\varvec{R}}$ is the residual variance–covariance matrix between $o$ traits. The model was implemented assuming $\user2{K }$ as unstructured matrix and ${\varvec{R}}$ as diagonal matrix (Lado et al. 2018; Arojju et al. 2020b) with 3000 iterations and a burn-in number of 1500 using the MTM R package (de los Campos and Grüneberg 2016).

**Fig. 1:**
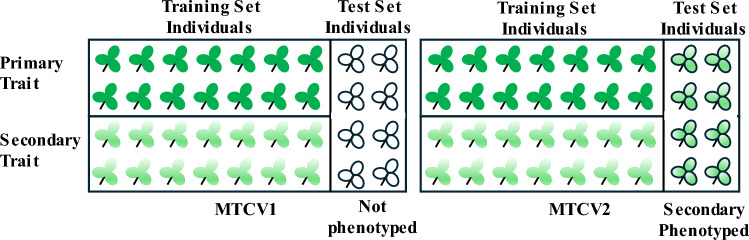
Cross-validation (CV) schema used for multi-trait (MT) genomic prediction representing two scenarios: MTCV1, where each individual in the training set is phenotyped for both primary and correlated secondary traits and the test set has no phenotype information when predicting GEBVs for the primary trait, and MTCV2, where each individual in the training set is phenotyped for both primary and secondary traits, and additionally the test set is phenotyped for the secondary trait when predicting GEBVs for the primary trait

Two different cross-validation (CV) schemes were implemented to assess multi-trait (MT) genomic prediction models as reported previously (Fernandes et al. 2018; Arojju et al. 2020b), representing scenarios encountered in a breeding programme. The first scheme is a multi-trait CV1 (MTCV1) (Fig. 1), where the training set is phenotyped for both the primary and the correlated secondary trait with the aim to predict the GEBVs for the primary trait in the test set, assuming the test set is not phenotyped for the primary nor the secondary trait. The second scenario is multi-trait CV2 (MTCV2), in which the training set is phenotyped for both primary and correlated secondary traits, and test set individuals are phenotyped for the secondary trait. These data are then incorporated into the prediction model to estimate GEBVs in the test set for the primary trait (Fig. 1). Performance of models was evaluated through Monte Carlo cross-validation, where 80% of HS families were randomly assigned to the training set and used to predict the remaining 20% of the test set.

### Population structure

Multi-dimensional scaling (MDS) was applied to the KGD-GRM to determine the presence of any structure in the population using the ‘cmd scale’ function in R and the first two dimensions were visualized using R (R Core Team 2018).

### Genetic gain prediction for dry matter yield

Deterministic modelling was performed using DeltaGen (Jahufer and Luo 2018) to compare predicted genetic gain achieved using a combined approach of among-HS family phenotypic selection and within-HS family selection via genomic selection (ApWFgs) to a common selection strategy used by forage breeders based on among-HS family phenotypic selection alone (HSp). Simulations were performed using 2 and 3 years of spring DMY data separately to assess the improvements in predicted genetic gain using additional phenotypic assessment. The spring DMY estimates of family, family × location and family × year variance components from Eq. 3 (Ehoche et al. 2022) were used to simulate the predicted genetic gain using HSp and ApWFgs breeding strategies. The base selection strategy assumed for both phenotypic (HSp) and genomic selection (ApWFgs) was 200 HS families evaluated for spring DMY by means of full cuts across three replicates in two environments. An extra year was added per breeding cycle for crossing and population establishment. Genetic gain per breeding cycle was compared at four different selection pressures (described below) using among-family selection (AFS) and within-family selection (WFS) breeding strategy.

**Fig. 2:**
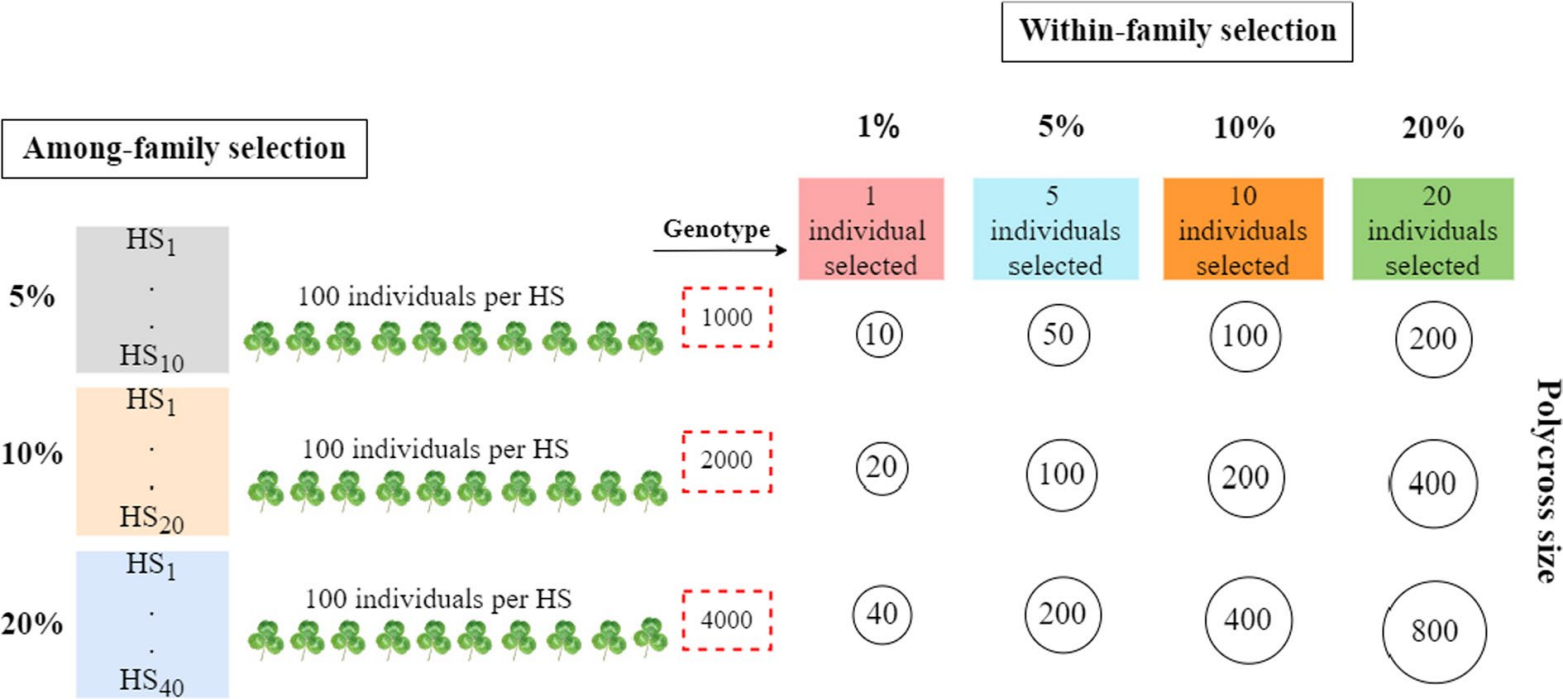
Selection scheme showing among- and within-family selection pressures for a population of 200 half-sib (HS) families and the resultant polycross sizes. Numbers in the red dashed box indicate the total number of individuals genotyped for that among-family selection pressure. For example: with 200 HS families, 5% among-family selection pressure = 10 HS families; genotyping 100 individuals from each of the 10 selected HS families = 1000 plants genotyped. Within-family 1% selection pressure with 100 plants genotyped per HS family = 1 individual selected per HS family; as there are 10 HS families at the 5% among-family selection pressure, there will be 10 genotypes (1 per HS family) selected for a 5% among-family and 1% within-family polycross

Different subsets of HS families were selected based on three AFS pressures of 20%, 10% and 5%. It was assumed that from each selected HS family, a random sample of 100 seedlings were established and genotyped to estimate GEBVs. Either of four WFS pressures were applied at 20%, 10%, 5% and 1% to select individuals with the highest GEBVs. As expected with increasing selection pressure, the number of selected individuals decreases. As the number of seedlings grown for each selected HS family was set at 100, if the number of HS families selected at the AFS stage is *y*, the total number of individuals genotyped at the WFS stage would be 100 × *y* (Fig. 2). Therefore, the same cost for genomic selection would apply across all WFS pressures under the same AFS pressure.

Genetic gain (*ΔG*) per cycle for HSp (Eq. 4) as described by Casler and Brummer (2008) and modified for among- and within-family selection (ApWFgs; Eq. 5), respectively, was calculated using DeltaGen (Jahufer and Luo 2018) as follows:

4 $$\Delta G_{{{\text{HS}}}} = k_{f} c \frac{{\frac{1}{4}\sigma_{A}^{2} }}{{\sigma_{PF} }}$$

where $\Delta G_{{{\text{HS}}}}$ is the genetic gain based on selection and random mating of the top performing HS families; $k_{f}$ is the among-family selection intensity; $c$ is the parental control; $\sigma_{A}^{2}$ is the additive variance; and $\sigma_{PF}$ is the among-family phenotypic standard deviation.

5 $$\Delta G_{AFp - WFgs} = k_{f} c_{f} \frac{{\frac{1}{4}\sigma_{AY}^{2} }}{{\sigma_{PF} }} + k_{W} c_{W} h_{X} r_{A - XY} \frac{\sqrt 3 }{2}\sigma_{PF}$$

where $\Delta G_{AFp - WFgs}$ is the genetic gain derived using a combination of among-family phenotypic selection and within-family selection via genomic selection (ApWFgs); $c_{f}$ and $c_{w}$ are the among- and within-family parental controls respectively; equal to 0.5 for HS families; $\sigma_{AY}^{2}$ is the additive genetic variance for the trait Y under selection; $k_{W}$ is the within HS family selection intensity; and $r_{A - XY}$ is the genomic selection predictive ability assumed as 0.3 and the square root of heritability of trait *X* given as $h_{X} .$ The predictive ability (0.3) was that obtained for spring DMY in this study.

## Results

### Genomic prediction models performed similarly

**Fig. 3:**
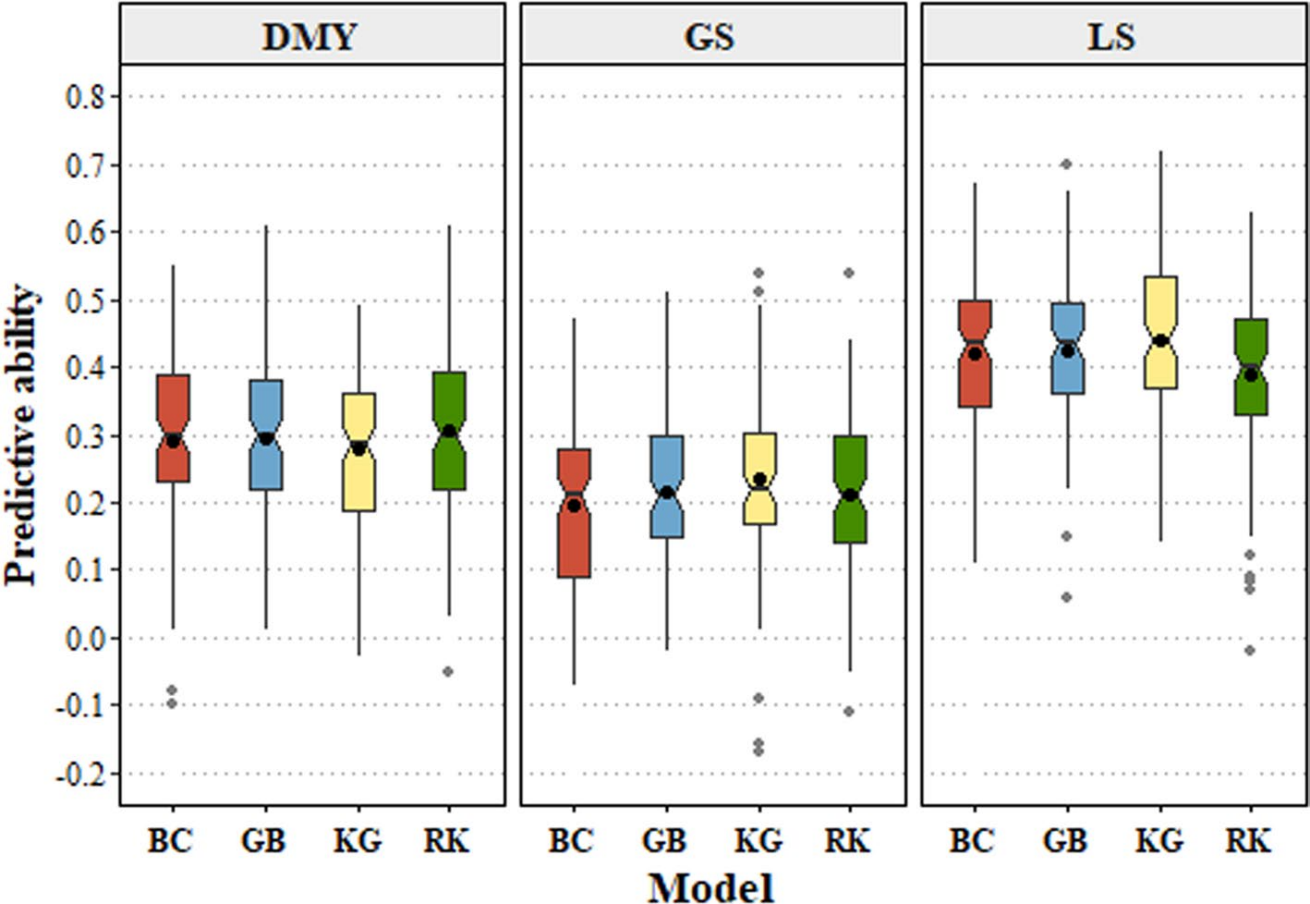
Comparison of predictive ability among single-trait genomic prediction models BayesCπ (BC), GBLUP (GB), KGD-GBLUP (KG) and RKHS (RK) for spring DMY (dry matter yield), GS (growth score) and LS (leaf size) combined across sites and years. Mean predictive ability was calculated based on 100 iterations using 80% of the data as training and 20% as test sets. Solid line represents the median, black dot in the box represents the mean, and grey dots are outliers. Notches that do not overlap indicate medians that are significantly different at *P* < 0.05 (Chambers et al. 1983)

No population structure was observed in the training population as depicted by the absence of clusters in the MDS plot in Fig. S1. Predictive ability across models ranged from 0.28 to 0.31 for spring DMY, 0.2 to 0.25 for GS and 0.41 to 0.44 for LS, based on BLUPs estimated by combining across sites and years data (Fig. 3). For spring DMY, the mean predictive ability of RKHS was slightly higher than BayesCπ, KGD-GBLUP and GBLUP, while KGD-GBLUP trended towards better performance than the other models for GS and LS, although these differences were not statistically significant (Fig. 3). For all traits, BayesCπ and RKHS had bias values closer to 1, while the bias values for KGD-GBLUP and GBLUP model were slightly inflated for all three traits ranging from 1.5 to 3.2 (Tables S1, S2). In summary, the prediction models performed similarly, and therefore, subsequent analysis over a range of traits was performed using KGD-GBLUP due to its efficient compute requirements and ability to handle low read-depth genotypes and missing data.

### Trait predictive ability increased with heritability

The narrow-sense heritability for various traits investigated in this study was reported previously by Ehoche et al. (2022). When assessing prediction models for LS, GS, spring DMY and stolon traits, there was a significant (*P* < 0.001) relationship between predictive ability and trait heritability, with heritability explaining a moderate proportion (*R*^2^ = 0.35) of the predictive ability (Fig. S2). Therefore, traits with higher heritability tended to have higher predictive ability. The highest predictive ability achieved was 0.44 for LS based on across-location and years phenotypic data which had the highest narrow-sense heritability. The lowest predictive ability of −0.17 was obtained for SBPRS Year 3.

### Predictive ability tended to increase with trial maturity

**Fig. 4:**
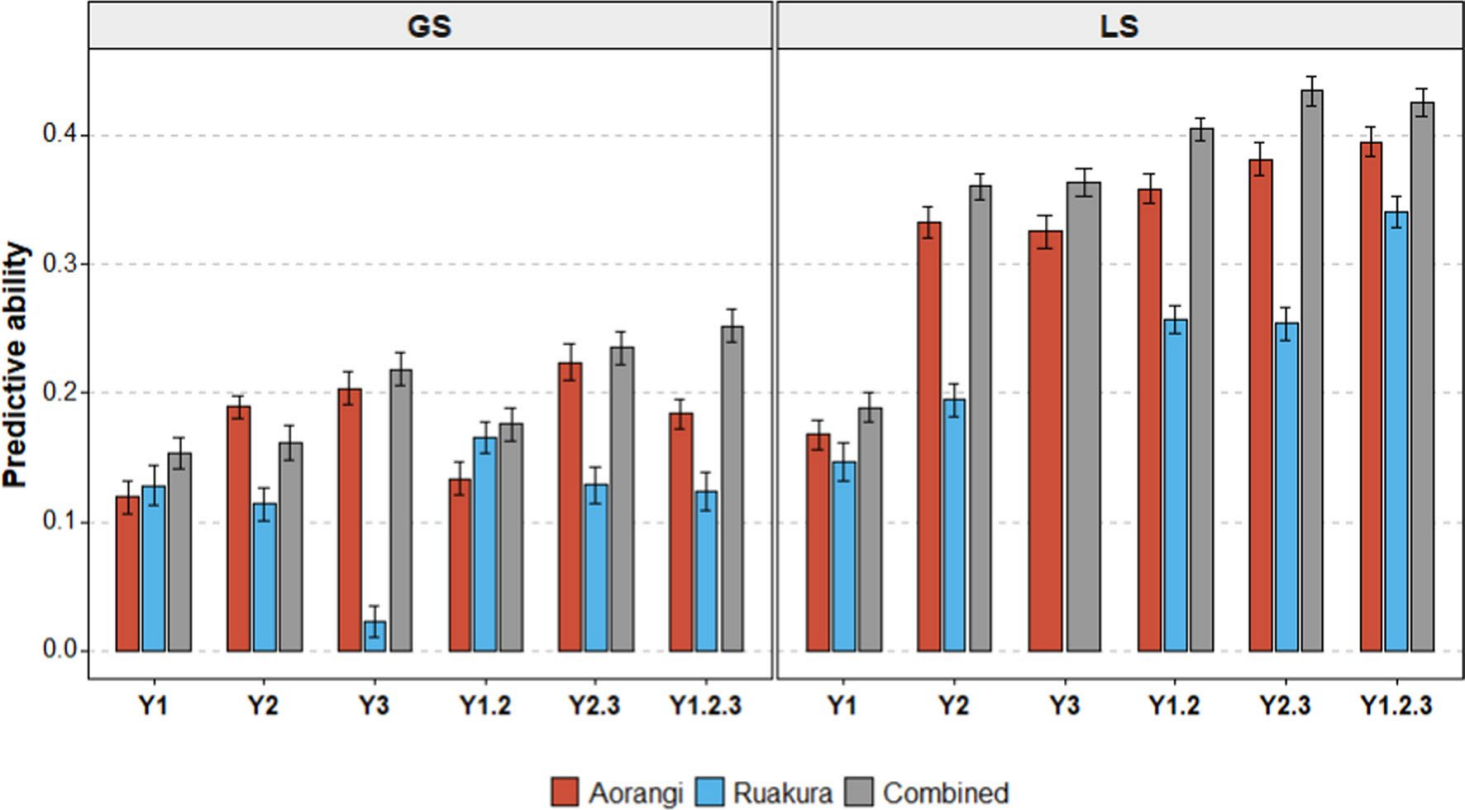
Predictive abilities for growth score (GS) and leaf size (LS) in two locations; Aorangi, Ruakura and Combined across-locations over a period of three individual years (Y1, Y2, Y3) or combinations of years (Y1.2, Y2.3, Y1.2.3). Models were derived using KGD-GBLUP for 100 iterations and predictive ability estimated using Monte Carlo cross-validation using 80% training, 20% test sets. Error bars represent standard errors of the mean

Predictive ability for GS and LS individually or across combinations of locations and years tended to increase with trial maturity. The highest predictive ability was obtained using phenotypic data from the 3rd year (Fig. 4) (Table S1). The exception to this was GS at Ruakura, which was constant for years 1 and 2, then dropped to 0.02 in year 3 (Fig. 4). For traits GS and LS, the phenotypic data combined across increasing years resulted in the highest predictive ability, compared to the predictive ability derived from individual years. The highest predictive ability for GS was 0.25 when data from all 3 years were combined. For LS, the predictive ability was similar (0.43) when the phenotypic data from years 2 and 3 and years 1, 2 and 3 were combined (Fig. 4). There was an increase in predictive ability for LS, from 0.17 in the 1st year to 0.43 when all 3 years were combined. Similarly, combining data from all 3 years for GS increased the predictive ability by 127% from the first-year predictive ability.

The predictive ability obtained in Ruakura was noticeably lower than in Aorangi. For the GS trait, the predictive ability ranged from 0.02 to 0.17 in Ruakura, while in Aorangi predictive ability was slightly higher with a range of 0.06 to 0.22 (Fig. 4). Similar results were obtained for LS, where the difference in average predictive ability between Aorangi and Ruakura was 40% (Fig. 4) (Table S1). Due to poor predictive ability, bias estimated for traits in Ruakura tended to have higher values (Table S1).

### Minimum training population size across traits determined to be 100 to 120

**Fig. 5:**
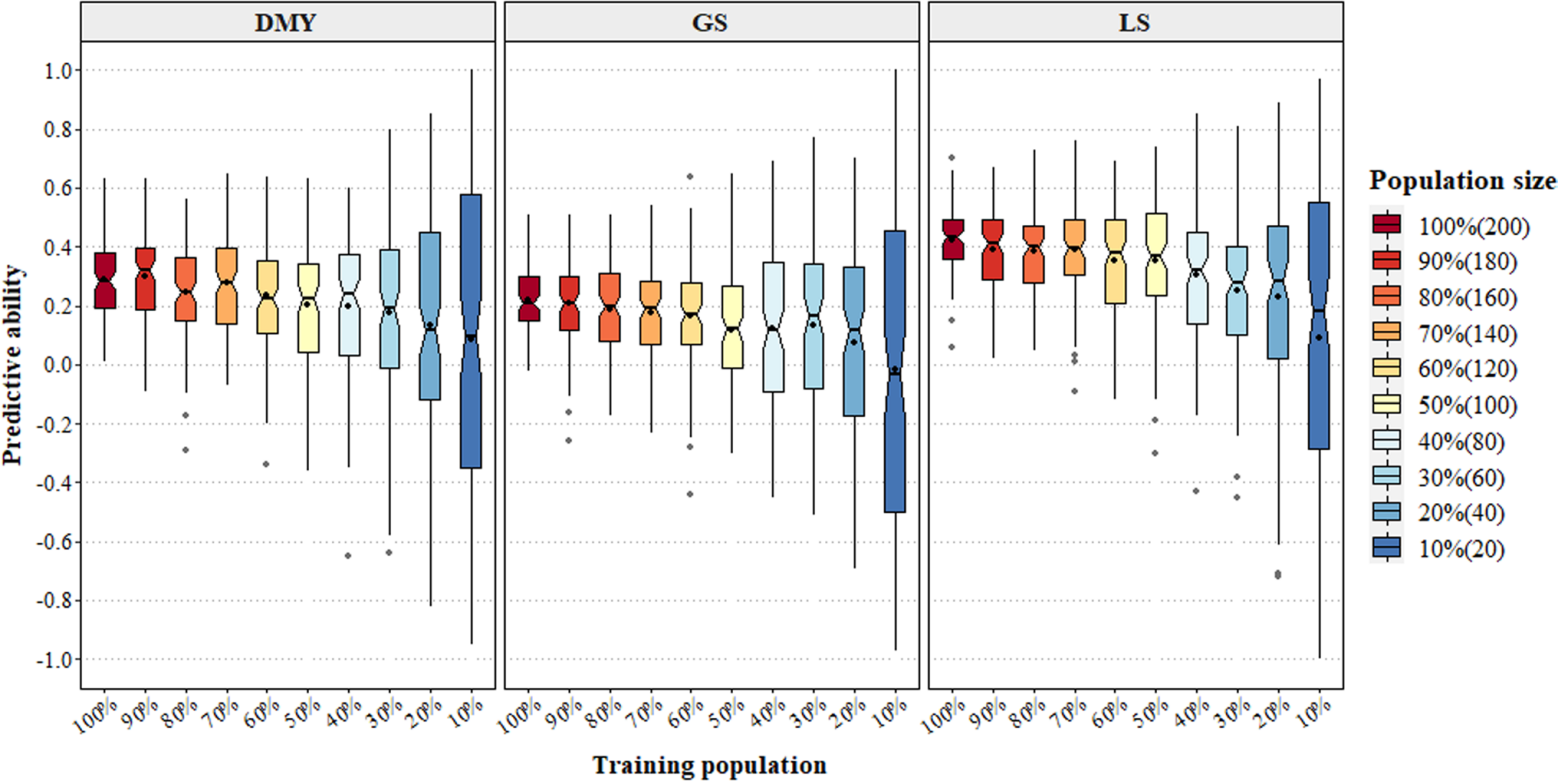
Notched boxplots of the effect of training set size on the predictive ability of three traits: spring DMY (dry matter yield), GS (growth score) and LS (leaf size) combined across sites and years. Model was run for 100 iterations using GBLUP. Predictive ability was estimated using Monte Carlo cross-validation with 80% training, 20% test sets. Solid line represents the median, black dot in the box represents the mean, and grey dots are outliers. Notches that do not overlap indicate medians that are significantly different at *P* < 0.05 (Chambers et al. 1983)

The influence of training population size on predictive ability was investigated and showed no significant difference in the predictive ability for LS when the training population size was reduced from 200 to 100 HS families (Fig. 5). A 28% reduction in predictive ability was only realized when the number of HS families was dropped to 80. The differences in predictive ability were more evident for the more complex trait spring DMY, as significant differences in predictive ability were observed when 120 HS families or less were used to train the model (Table S3). Predictive ability for spring DMY using all HS families was 0.3 and dropped to 0.2 and 0.1 when 50 and 20 individuals were used to train the model, respectively. For the GS trait, significant differences in predictive ability were observed when 100 or fewer HS families were used to train the model. Using training sets above 120 or more families for GS resulted in a similar predictive ability to that when using all 200 individuals. In general, there was also an increase in variation in the predictive ability when fewer individuals were included in the model (Fig. 5). The variation in bias tended to be higher in reduced training set sizes (Table S3). In summary, across a range of simpler and complex traits, it appears that for this training population, a minimum of 100 to 120 individuals was sufficient to generate predictive abilities similar to the full population.

### A set of 5500 SNP markers was sufficient for genomic prediction modelling

**Fig. 6:**
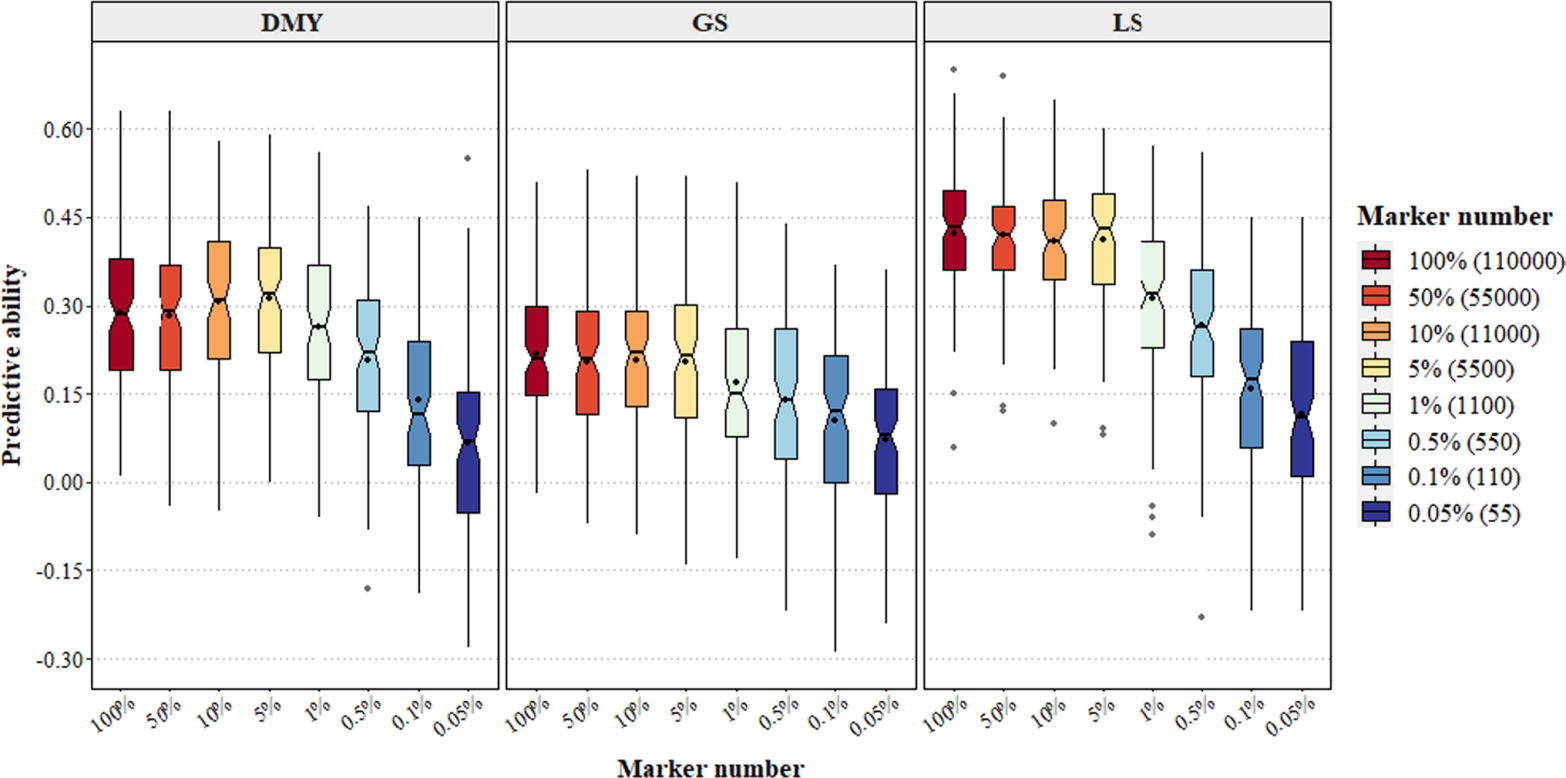
Notched boxplots of the effect of marker number on the predictive ability of three traits: spring DMY (dry matter yield), GS (growth score) and LS (leaf size) combined across sites and years. Model was run for 100 iterations using GBLUP. Predictive ability was estimated using Monte Carlo cross-validation with 80% training, 20% test sets. Solid line represents the median, black dot in the box represents the mean, and grey dots are outliers. Notches that do not overlap indicate medians that are significantly different at *P* < 0.05 (Chambers et al. 1983)

No significant gain in predictive ability was observed when more than 5% (5500) of markers were used for LS, GS and DMY (Fig. 6). The predictive ability for spring DMY began to reduce from 5550 markers or less, as evidenced by a decrease in predictive ability from 0.29 when using all markers to 0.19 at 550 markers. For GS, the predictive ability was consistent at 0.21 when markers were reduced from 110,000 to 5500. However, when the markers used for constructing the GRM were reduced stepwise from 5500 to 55, the predictive ability dropped progressively to 0.06. Similarly, the predictive ability for LS reduced markedly from 0.42 to 0.31 when only 1100 markers were used. Reducing marker set size did not seem to increase the sampling variation as evidenced from the boxplots (Fig. 6). Also, reducing the number of markers generally increased the bias for spring DMY and GS, whereas for LS this metric was stable from 1% to larger marker sets (Table S4). It appears that with this population, a marker set 5500 or greater was sufficient to generate predictive abilities similar to the full marker set of 110,000.

### Incorporating correlated traits can increase predictive ability for a primary trait

**Fig. 7:**
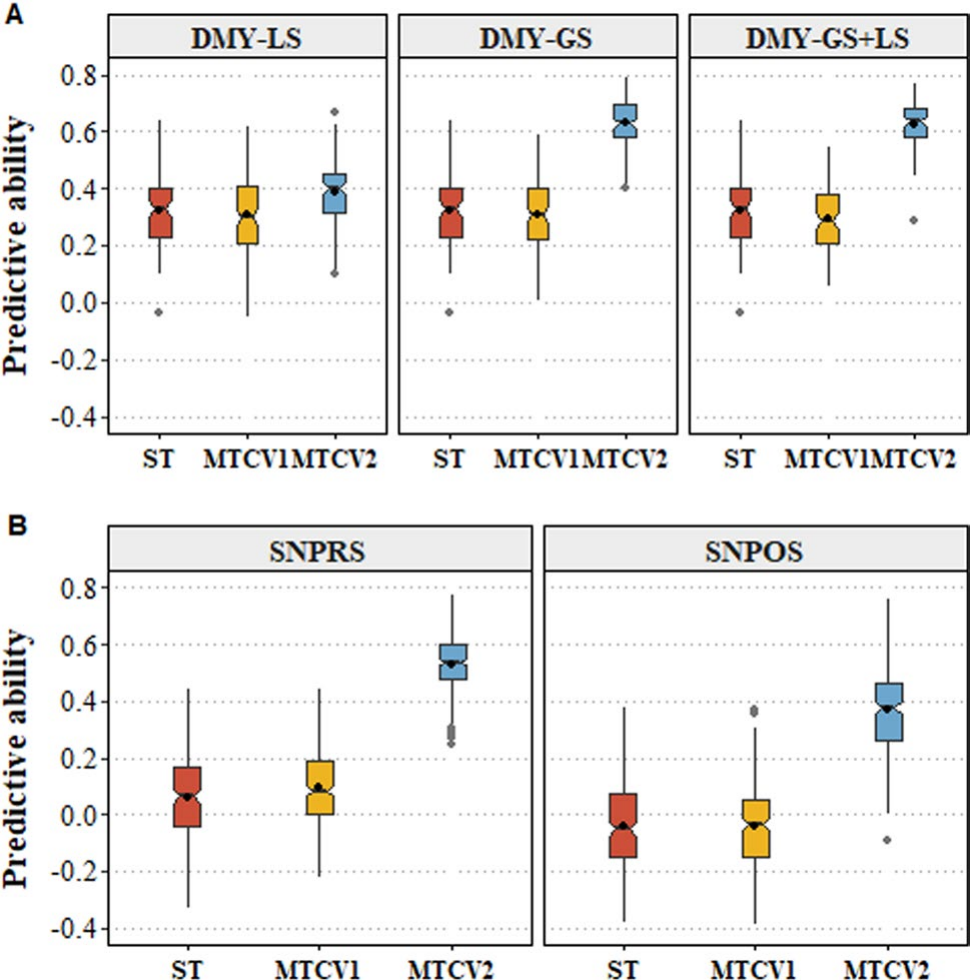
**A** Comparison of the predictive ability of single-trait model (ST) and multi-trait model for spring dry matter yield combined across sites and years (DMY). In multi-trait models, growth score (GS) and leaf size (LS), combined across sites and years, were used as secondary traits correlated to DMY, the primary trait. **B** Comparison of the predictive ability of single-trait model for pre-summer (SNPRS) and post-summer (SNPOS) stolon number when incorporating secondary traits, pre-summer (SBPRS) and post-summer (SBPOS) stolon branching. Cross-validation schemes, multi-trait cross-validation 1 (MTCV1), which corresponds to predicting untested phenotypes in the test set and MTCV2 which predicts breeding values for the primary trait in the test set, based on information from the correlated secondary traits, were implemented

As described previously (Ehoche et al. 2022), the correlation between spring DMY and the GS trait was positive and high (pairwise Pearson correlation coefficient of 0.73), and a moderate correlation (0.35) was observed between the spring DMY and LS. There was also a positive correlation of 0.67 between pre-summer SNPRS and SBPRS stolon traits, and for post-summer SNPOS and SBPOS traits the correlation was 0.63 (Ehoche et al. 2022). The predictive ability of the MTCV1 approach for spring DMY, when incorporating either GS or LS, or both, as secondary traits with phenotype values in the training set only, was no different to the single-trait genomic prediction model (Fig. 7A). By contrast, the MTCV2 approach, based on phenotyping the primary trait in the training set only and the correlated secondary trait information (single or multiple traits) in both training and test individuals, increased predictive ability by 28% for spring DMY-LS and 124% for spring DMY-GS, respectively (Fig. 7A). The percentage increase in predictive ability was proportional to the degree of correlation between the primary and secondary trait. The multi-trait approaches (MTCV1 and MTCV2), which include both GS and LS as secondary traits to predict spring DMY (spring DMY-GS + LS), did not improve predictive ability over spring DMY-GS (Fig. 7A). For complex stolon-related traits, the results were similar to spring DMY, where MTCV1 did not outperform the single-trait genomic prediction model (Fig. 7B). In the MTCV2 approach, predictive ability for SNPRS and SNPOS increased from 0.15 to 0.54 and −0.1 to 0.28, using the secondary traits SBPRS and SBPOS (Fig. 7B). The bias values obtained from MTCV1 and MTCV2 were closer to 1, suggesting an unbiased prediction from the multi-trait prediction model (Table S5).

### Modelling shows genomic selection increases genetic gain for dry matter yield

**Fig. 8:**
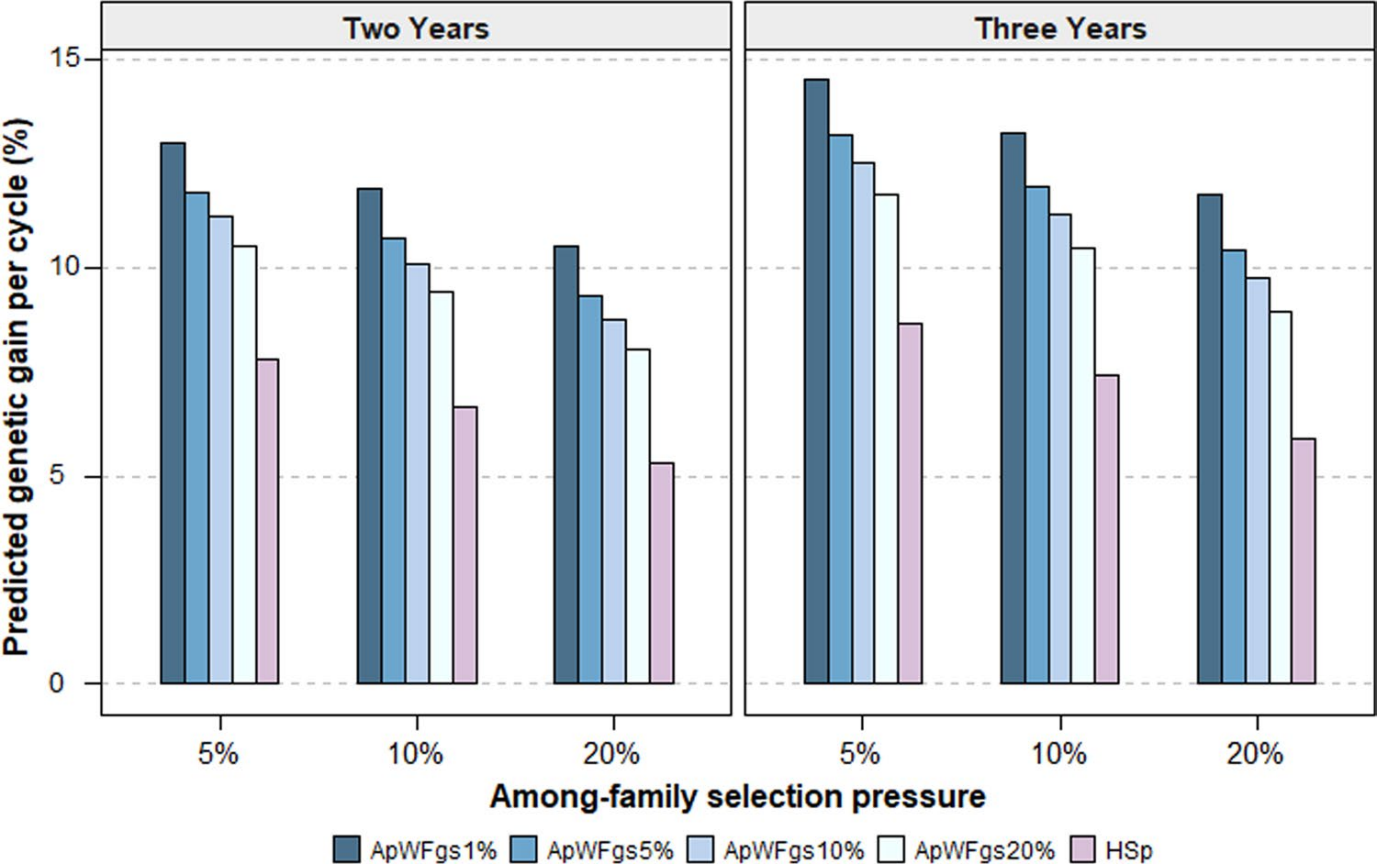
Predicted genetic gain per cycle for combined multi-site multi-year spring dry matter yield (DMY) for among-half-sib family phenotypic selection (HSp) compared with a breeding strategy of among-family phenotypic selection and within-family genomic selection (ApWFgs) at different selection pressures. Among-family selection pressures were combinations of 5%, 10% and 20%, and within-family selection pressures were, 1%, 5%, 10% and 20%. Results were based on spring DMY data from 2 and 3 years for 200 half-sib families evaluated in two locations

Using a traditional among-HS family phenotypic selection (HSp) breeding strategy based on two years of multi-site spring DMY data, the predicted genetic gain was 5.3%, 6.7% and 7.8% per selection cycle at an among-HS family selection (AFS) pressure of 20%, 10% and 5%, respectively (Fig. 8). Predicted genetic gain increased for this strategy when using 3 years of spring DMY (5.9%, 7.4% and 8.7%) compared to 2 years of data (Fig. 8). A combined among-HS family phenotypic selection and within-HS family selection via genomic selection (ApWFgs) breeding strategy gave a marked increase in predicted genetic gain per cycle (Fig. 8). Using genomic selection to select best plants within the best families (WF_gs_) increased predicted genetic gain per cycle by at least 31% relative to HSp strategy at the various selection pressures. Combining 3 years of multi-site spring DMY data gave greater predicted rates of genetic gain than 2 years of data. The highest predicted genetic gain was observed using 1% within-family selection pressure at various among-family selection pressures (20%, 10% and 5%) (Fig. 8).

## Discussion

Here, we report the first study investigating the feasibility of implementing genomic selection in white clover by exploring aspects of different models, population size, marker density and trait architecture on predictive ability. Furthermore, we estimated the influence of genomic selection by comparing the predicted genetic gains for spring DMY in two different breeding strategies: a traditional among-HS family phenotypic selection (HSp) and incorporating genomic selection using among-HS family phenotypic selection and within-HS family genomic selection (ApWFgs).

### Genomic selection in white clover

We investigated four single-trait genomic prediction models for spring DMY, GS and LS traits, both individually and across different years and locations. Our findings indicated that each model achieved comparable predictive abilities for the respective traits. Similarly, studies by Arojju et al. (2018) and Roorkiwal et al. (2016) found no significant improvements in predictive ability when comparing different models in ryegrass (*Lolium perenne*) and chickpea (*Cicer arietinum*), respectively. Traditionally, LS is considered to have a qualitative inheritance pattern controlled by few genes with larger genetic effects (Ashri 1968). For such traits, variable selection models like BayesCπ have been shown to outperform the GBLUP model (Daetwyler et al. 2013). Contrary to these expectations, the predictive ability for LS based on the BayesCπ model was similar to the KGD-BLUP and GBLUP models in the current study. Since there was no significant difference in predictive ability among different genomic prediction models in our training population, considering the computational time for model development, KGD-GBLUP was selected as the most suitable for implementing genomic selection in this white clover training population.

In this study, for traits influenced by high G × E, such as spring DMY, GS and stolon branching traits, as detailed in Ehoche et al. (2022), predictive ability and heritability were relatively low. There was, however, a positive correlation between predictive ability and trait heritability, which aligns with previous findings by Arojju et al. (2018) in ryegrass and Skøt et al. (2024) in red clover. This relationship has been corroborated further through simulations where trait predictive ability decreased as quantitative trait loci (QTL) number, a proxy for trait complexity, increased (Zhong et al. (2009). Another approach to increasing predictive ability for low-heritability traits is utilizing larger training population sizes (Meuwissen et al. 2001; Goddard and Hayes 2007). However, establishing such a large training population in forages may be impractical due to various limitations including the challenges of generating and maintaining these extensive populations as well as the complexities of phenotyping of multi-site trials on this scale. These issues will lead to a substantial burden in terms of financial and other resource requirements. Alternative approaches like multi-trait genomic prediction (Arojju et al. 2020b) or pooling multiple breeding populations can be employed to build large training populations, which will be discussed in the later part of this section.

There was a general trend towards improved predictive ability as the trial matured or when data from multiple years and locations were combined. Similarly, Grinberg et al. (2016) noted an increased predictive ability in perennial ryegrass in the second year compared to the first year’s data. Spindel et al. (2015) also found predictive ability to increase when data from all years were used to train genomic prediction models in rice. In the current study, the increase in predictive ability in the second and third year compared to the first can be explained by the higher amount of additive genetic variation estimated in those years due to more confounding factors being accounted for. This may also reflect variability in the establishment phase and the transition from a tap-rooted seedling to the stoloniferous plant that occurs approximately 18 months after planting. Due to the considerable phenotypic changes that occur during this transition, we therefore suggest that clover trials be run for a minimum of 3 years to collect sufficient data at the mature stoloniferous growth form which underpins persistence in the sward (Ehoche et al. 2022; Woodfield and Caradus 1996).

With respect to multi-location data, Faville et al. (2018) and Annicchiarico et al. (2015) obtained higher predictive ability when data were pooled across environments in ryegrass and alfalfa, respectively. Skøt et al. (2024) found predictive ability to be higher when using a model that incorporated marker × environment interaction effects versus a single-environment model. Multi-year and multi-location data can capture more variation and effectively separate G × E from additive variation, while single year or single location data are possibly confounded by G × E effects, causing a reduction in predictive ability. Utilizing models developed from multi-location data is, therefore, usually preferable to a single location model when G × E is substantial.

While the size of the training population was modest at 200 HS families, the trial represented a significant use of resources as it comprised 1344 plots after inclusion of repeated checks, replication within and among locations (Ehoche et al. 2022). We assessed the influence of reducing the number of HS families used to train the prediction model. For all traits, the predictive abilities derived from 100 to 120 individuals were similar to the complete training population of 200 HS families. There was, however, an increase in the variability of the predictive ability during cross-validation as the size of the training population decreased. This variability was acceptable between 100 and 200 families but became more pronounced as the population size dropped below 100. This indicates larger training populations may enable more accurate prediction of GEBVs, but that 100 to 120 entries are an effective minimum for complex white clover traits assessed in multi-year multi-site field trials. Previous studies have shown increases in training population size leads to increased predictive ability, as the additional information enables maker effects to be precisely estimated with less variability (Resende et al. 2012). In forages, assembling larger training populations will remain a challenge due to logistical constraints described above. However, genomic selection can also be implemented by assembling multiple breeding populations as a single training set to increase the training population size. This approach was demonstrated by Schulz-Streeck et al. (2012) in maize and Faville et al. (2018) in perennial ryegrass. This would be a similar scenario to that which has been used in animal breeding by assembling multi-breed training populations to implement genomic selection.

Marker number is a key component of genomic prediction models. Developing high-density marker resources such as SNP chips is costly and subject to ascertainment bias as the SNPs identified are relevant only to the populations in which the SNPs were validated (Albrechtsen et al. 2010). Therefore, for many forage and crop species, platforms such as genotyping by sequencing (GBS) (Elshire et al. 2011) are an option for implementing genomic selection. A total of 110,000 GBS SNPs were identified and used for genomic prediction in the current white clover training population. Reducing the number of markers had little influence on predictive ability as similar predictions and prediction variabilities were achieved with significantly fewer markers (5%; *n* = 5500). Similarly, Skøt et al. (2024) found that predictive ability did not significantly decrease when the number of markers was reduced from 20,000 to 1000. This suggests that the genomic relationship matrix describing the training population and incorporated into the GBLUP model was accurately captured using 5500 SNPs. In addition, when the training and breeding population are closely related, as in this situation, a large proportion of accuracy is due to the relatedness captured by the markers (Goddard et al. 2011; Kainer et al. 2015; Arojju et al. 2018). Genotyping costs can hinder implementation of genomic selection in a breeding programme by influencing the number of individuals sequenced, as well as the sequencing depth which contributes to the resulting number of GBS markers (Li et al. 2011). Gorjanc et al. (2017) demonstrated that even at relatively low depth (< 2×), genotyped markers were able to provide comparable prediction accuracies thus providing a robust, cost-effective approach suitable for implementing genomic selection. In summary, low-coverage genotyping platforms like GBS, which can produce large numbers of markers at low depth, coupled with genomic prediction models like KGD-GBLUP designed to utilize low read-depth sequence data (Dodds et al. 2015), would be a suitable and flexible option for implementing genomic selection in white clover breeding programmes.

The goal of multi-trait genomic selection is to take advantage of correlated secondary trait information to improve the predictive ability of a more expensive and difficult-to-measure trait (Jia and Jannink 2012). In our study where we investigated the MTCV1 approach, which assumes a scenario of predicting untested phenotypes for the primary trait by incorporating a secondary trait information in the prediction model. There was, however, no significant boost in predictive ability detected. This observation is consistent with results from several studies (Fernandes et al. 2018; Velazco et al. 2019a; Ward et al. 2019). However, in the MTCV2 approach where the training set was phenotyped for both primary and correlated secondary traits and test set individuals were phenotyped for the secondary trait, there was a significant increase in predictive ability for spring DMY and SNPRS. Osterman et al. (2024) also observed an improvement in predictive ability with multi-trait models for traits with moderate to high genetic correlations. Wang et al. (2017) and Velazco et al. (2019a) reported that including multiple secondary traits in the MTCV2 approach also improved the predictive ability for the primary trait. By contrast, our results contradict this finding, as including both LS and GS as secondary traits in the multi-trait model did not significantly improve the predictive ability for spring DMY, compared to a single secondary trait (GS) in the model. The increase in predictive ability using MTCV2 approach results not only from the additional information obtained from the secondary trait, but also mostly due to the exploitation of the correlation between traits (Velazco et al. 2019a; Arojju et al. 2020b). This was evident for spring DMY as the increase in predictive ability was higher using the highly correlated trait GS, compared to low correlated trait LS. The applicability of the MTCV2 approach in a forage breeding programme has been described previously by Arojju et al. (2020b).

### Expected genetic gain for DMY using genomic selection

Conventional breeding in white clover is often based on an among-HS family (HSp) breeding strategy with limited ability to implement within-HS family selection (WFS). According to Casler (2008), the low rates of genetic gain in forage crops are primarily due to the inability to access within-family variation efficiently by the selection methods. Genomic selection can accurately predict the breeding values for candidates within family, which is reflected in the increased genetic gain per cycle of selection, as observed in this study through deterministic simulations. The maximum predicted genetic gain achieved through ApWFgs was almost twice that obtained by HSp breeding strategy. This was confirmed further through simulations by Lin et al. (2016), Jahufer et al. (2021) and Endelman et al. (2014) that genomic selection can substantially increase genetic gain compared to conventional breeding in ryegrass and maize. Ultimately, the uptake of genomic selection by breeding programmes will depend upon the cost per unit of genetic gain achieved. As the costs of genotyping continue to decline while the expenses associated with phenotyping are on the rise, genomic selection is poised to become a preferred breeding tool, enabling the implementation of WFS with greater selection intensity at a relatively lower cost compared to traditional phenotypic selection.

Maintaining genetic diversity is essential for long-term success of a recurrent breeding programme. Particularly in outcrossing species such as white clover, selections from populations with low genetic diversity can lead to detrimental effects due to high rates of inbreeding (Gorjanc et al. 2018). In our simulations, highest genetic gain was obtained with the highest selection pressure (AFS 5% and WFS 1%). There needs to be a trade-off in the percentage genetic gain in the initial selection cycles, to maintain the genetic diversity and low rate of inbreeding for the long-term success of the breeding programme. Considering that the rate of inbreeding could be relatively high when implementing genomic selection compared to conventional phenotypic selection (Lin et al. 2017; Bandillo et al. 2022), depending on the level of diversity in the population, lower selection pressures (AFS 20% and WFS 20%) for population improvement could be followed by higher selection pressures (AFS 5% and 1% WFS) during the cultivar development phase. This two-part strategy can be balanced easily through genomic selection by managing the number of genotyped individuals in each breeding cycle.

One of the well-established applications of genomic selection is to reduce the breeding cycle length by predicting GEBVs without a need for phenotyping the selection individuals (Bernardo 2002; Bassi et al. 2016). The deterministic simulations reported here estimated the genetic gain for spring DMY after one cycle of selection using both 2 and 3 years of data. When considering time per breeding cycle, genetic gain can be further advanced by implementing genomic selection to complete multiple breeding cycles within the same time period required to implement conventional phenotypic selection (Bernardo 2002; Bassi et al. 2016). Although a breeding cycle based on a HSp strategy takes at least 3 years, genomic selection can be used to perform up to two cycles of selection per year provided daylength and vernalization requirements are met.

While the predictive abilities identified across the traits in the current field trial data were modest (0.17–0.44), this can still facilitate genetic gain in complex traits in a HS family breeding scheme through reduced breeding cycle times, as described above, and through accessing the three quarters of additive variation that resides within families (Falconer 1989). These predictive abilities equated to prediction accuracies of 0.33–0.65 (Supplementary Table S1), and previous studies have shown that genomic selection performed as well or better than phenotypic selection for seed yield in soybean with prediction accuracies of 0.27–0.42 (Bandillo et al. 2022). This further supports that genomic selection can be deployed in the forage crops in white clover and made commercially viable through practicable numbers of families/trial plots and the availability of cost-effective marker platforms. However, the main purpose of genomic selection is to predict the performance of untested individuals, and the true performance of genomic prediction models must be validated empirically in breeding trials (Bassi et al. 2016; Ehoche et al. 2021). This next step is underway for white clover in this programme as synthetic populations have been developed based on these selection strategies and are currently being tested in field trials to empirically evaluate DMY genomic prediction.

## Conclusion

This study represents the feasibility of implementing genomic selection to improve DMY and persistence in white clover. The following conclusions were reached: (i) The genomic prediction models tested delivered similar predictive abilities despite having different underlying assumptions. (ii) Trait heritability was highly correlated with predictive ability and traits with higher heritability and low G × E reported higher predictive abilities, emphasizing the importance of good quality phenotypic data for implementing genomic selection. (iii) Similar predictive ability was achieved when one third of the training population was removed and only 5% of markers were used. High genotyping costs are a possible deterrent to the adoption of genomic selection in many breeding programmes, whereas low-depth genotyping coupled with the KGD-GBLUP model can reduce the cost for implementing genomic selection. Furthermore, our findings suggest that genomic prediction can be estimated using fewer families, which has significant impacts on multi-site multi-year trial size and subsequent feasibility for implementing genomic selection for forages. (iv) Multi-trait models substantially improved predictive ability highlighting potential for improved genetic gain of a primary trait through inclusion of a simple/inexpensive correlated secondary trait. (v) Based on the deterministic modelling, the integrated phenotypic selection and genomic selection approach, ApWFgs, resulted in higher predicted genetic gain over half-sib family phenotypic selection (HSp) despite a modest predictive ability of 0.3. This study confirms the potential advantages of integrating genomic selection with phenotypic selection to significantly increase genetic gain in white clover. Using cost-effective GBS paired with a prediction model optimized for low read-depth data, the approach can achieve prediction accuracies comparable to traditional models, providing a viable path for implementing genomic selection in white clover.

## Supplementary Information

Below is the link to the electronic supplementary material.

Supplementary file1 (DOCX 86 KB)

## Data Availability

Best linear unbiased predictors (BLUPs) phenotype data are available at Ehoche et al. 2022 (https://doi.org/10.1002/csc2.20793), and genotyping-by-sequencing data will be uploaded to a short-read archive repository upon acceptance of the manuscript (details to be confirmed). Further enquiries can be directed to the corresponding author.
