# Supplementary material for: Genomic selection shows improved expected genetic gain over phenotypic selection of agronomic traits in allotetraploid white clover
Source: Theor Appl Genet. 2025 Jan 23;138(1):34. doi: 10.1007/s00122-025-04819-w (PMC11757872; doi:10.1007/s00122-025-04819-w)
Supplement: Supplementary file 1 — Supplementary file1 (DOCX 86 KB) [file 122_2025_4819_MOESM1_ESM.docx]

Supplementary Information

Genomic selection shows improved expected genetic gain over phenotypic selection of agronomic traits in allotetraploid white clover.

O. Grace Ehoche^1,2,3^, Sai Krishna Arojju^1,4^, MZ Zulfi Jahufer^1^, Ruy Jauregui^1,5^, Anna C. Larking^1^, Greig Cousins^3^, Jennifer A. Tate^2^, Peter J. Lockhart^2^, Andrew G. Griffiths^1*^

^1^AgResearch Ltd, Grasslands Research Centre, Private Bag 11008, Palmerston North, New Zealand.

^2^Massey University, Private Bag 11222, Palmerston North, New Zealand.

^3^Current address: PGG-Wrightson Seeds, ℅ AgResearch Grasslands Research Centre, Palmerston North, New Zealand.

^4^Current address: Radiata Pine Breeding Company, Building EN27, University of Canterbury, Christchurch 8041

^5^Current address: Animal Health Lab, Ministry for Primary Industries. Wallaceville, New Zealand.

***Correspondence:** [andrew.griffiths@agresearch.co.nz](mailto:andrew.griffiths@agresearch.co.nz) ORCiD: 0000-0002-0573-1668

# Supplementary Figures


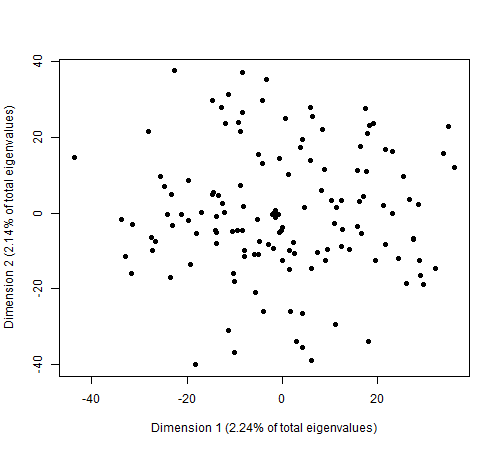
**Fig. S1** Multi-dimensional scaling (MDS) plot estimated from a genomic relationship matrix (GRM) of the 200 maternal parents of a 200 half-sib family training population. The GRM was calculated with 110,000 genotyping by sequencing-derived SNP markers.


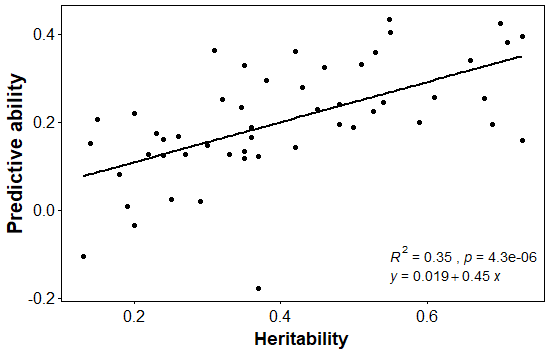


Fig. S2 Regression of predictive ability on narrow-sense heritability for multi-year, multi-site data for traits spring dry matter, leaf size, growth score, stolon number and stolon branches based on 200 half-sib families measured in 2017, 2018, 2019, in two locations, Aorangi and Ruakura New Zealand. Stolon branching and stolon number were measured in Aorangi only. Heritability data from Ehoche et al (2022).

**Supplementary Tables**

**Table S1:** Predictive ability, prediction accuracy and bias for measured traits with significant additive variation, determined using KGD-GBLUP.

| Trait | Location | Year | Heritability | Predictive ability | Prediction accuracy | Bias |
| --- | --- | --- | --- | --- | --- | --- |
| Spring DMY | Aorangi | 1 | 0.27 | 0.13 | 0.25 | 3.16 |
| Spring DMY | Aorangi | 2 | 0.48 | 0.24 | 0.35 | 1.68 |
| Spring DMY | Aorangi | 1.2 | 0.54 | 0.25 | 0.33 | 1.92 |
| Spring DMY | Aorangi | 1.2.3 | 0.45 | 0.23 | 0.34 | 6.97 |
| Spring DMY | Across sites | 1 | 0.37 | 0.12 | 0.2 | 2.13 |
| Spring DMY | Across sites | 2 | 0.35 | 0.33 | 0.56 | 1.46 |
| Spring DMY | Across sites | 1.2 | 0.38 | 0.3 | 0.48 | 1.73 |
| Spring DMY | Across sites | 1.2.3 | 0.43 | 0.28 | 0.43 | 2.06 |
| GS | Aorangi | 1 | 0.35 | 0.12 | 0.2 | 4.55 |
| GS | Aorangi | 2 | 0.50 | 0.19 | 0.27 | 2.5 |
| GS | Aorangi | 1.2 | 0.35 | 0.13 | 0.23 | 6.16 |
| GS | Aorangi | 3 | 0.59 | 0.2 | 0.27 | 1.66 |
| GS | Aorangi | 2.3 | 0.53 | 0.22 | 0.31 | 2.8 |
| GS | Aorangi | 1.2.3 | 0.48 | 0.2 | 0.28 | 2.55 |
| GS | Ruakura | 1 | 0.33 | 0.13 | 0.22 | 6.77 |
| GS | Ruakura | 2 | 0.42 | 0.14 | 0.22 | 7.76 |
| GS | Ruakura | 1.2 | 0.36 | 0.17 | 0.28 | 2.75 |
| GS | Ruakura | 3 | 0.29 | 0.02 | 0.04 | 0.38 |
| GS | Ruakura | 2.3 | 0.22 | 0.13 | 0.27 | 5.11 |
| GS | Ruakura | 1.2.3 | 0.73 | 0.16 | 0.19 | 4.17 |
| GS | Across sites | 1 | 0.24 | 0.15 | 0.26 | 2.12 |
| GS | Across sites | 2 | 0.24 | 0.16 | 0.33 | 4.02 |
| GS | Across sites | 1.2 | 0.23 | 0.18 | 0.37 | 2.26 |
| GS | Across sites | 3 | 0.20 | 0.22 | 0.49 | 1.88 |
| GS | Across sites | 2.3 | 0.35 | 0.23 | 0.4 | 2.33 |
| GS | Across sites | 1.2.3 | 0.32 | 0.25 | 0.45 | 3.2 |
| LS | Aorangi | 1 | 0.26 | 0.17 | 0.33 | 1.48 |
| LS | Aorangi | 2 | 0.51 | 0.33 | 0.47 | 1.77 |
| LS | Aorangi | 1.2 | 0.53 | 0.36 | 0.49 | 1.79 |
| LS | Aorangi | 3 | 0.46 | 0.33 | 0.48 | 1.22 |
| LS | Aorangi | 2.3 | 0.71 | 0.38 | 0.45 | 1.56 |
| LS | Aorangi | 1.2.3 | 0.73 | 0.39 | 0.46 | 1.72 |
| LS | Ruakura | 1 | 0.30 | 0.15 | 0.27 | 0.88 |
| LS | Ruakura | 2 | 0.69 | 0.19 | 0.23 | 1.39 |
| LS | Ruakura | 1.2 | 0.61 | 0.26 | 0.33 | 1.19 |
| LS | Ruakura | 2.3 | 0.68 | 0.25 | 0.31 | 1.33 |
| LS | Ruakura | 1.2.3 | 0.66 | 0.34 | 0.42 | 1.45 |
| LS | Across sites | 1 | 0.36 | 0.19 | 0.31 | 0.93 |
| LS | Across sites | 2 | 0.42 | 0.36 | 0.56 | 1.61 |
| LS | Across sites | 1.2 | 0.55 | 0.4 | 0.55 | 1.47 |
| LS | Across sites | 3 | 0.31 | 0.36 | 0.65 | 1.43 |
| LS | Across sites | 2.3 | 0.55 | 0.43 | 0.59 | 1.69 |
| LS | Across sites | 1.2.3 | 0.70 | 0.44 | 0.51 | 1.63 |
| Stolon branches (POS) | Aorangi | 1 | 0.15 | 0.21 | 0.54 | 2.3 |
| Stolon branches (POS) | Aorangi | 2 | 0.19 | 0.01 | 0.02 | -18.3 |
| Stolon branches (PRS) | Aorangi | 1 | 0.37 | -0.18 | -0.29 | NA |
| Stolon branches (PRS) | Aorangi | 2 | 0.18 | 0.08 | 0.19 | 21.3 |
| Stolon number (POS) | Aorangi | 1 | 0.25 | 0.02 | 0.05 | 0.29 |
| Stolon number (POS) | Aorangi | 2 | 0.13 | -0.11 | -0.29 | NA |
| Stolon number (PRS) | Aorangi | 1 | 0.20 | -0.03 | -0.08 | NA |
| Stolon number (PRS) | Aorangi | 2 | 0.14 | 0.15 | 0.41 | 0.9 |

**Table S2:** Effect of Genomic selection model on the predictive ability (PA), bias and bias range for traits dry matter (DMY), growth score (GS) and leaf size (LS) combined across sites and years.

| **Trait** | **Model** | **PA** | **Bias** | **Bias range** |
| --- | --- | --- | --- | --- |
| Spring DMY | BayesCπ | 0.29 | 1.08 | -0.74 - 3.46 |
| GS | BayesCπ | 0.20 | 0.80 | -0.21 - 2.05 |
| LS | BayesCπ | 0.42 | 1.41 | 0.35 - 2.54 |
| Spring DMY | GBLUP | 0.29 | 2.17 | 0.04 – 11.28 |
| GS | GBLUP | 0.21 | 2.36 | -0.17 - 15.65 |
| LS | GBLUP | 0.42 | 1.59 | 0.22 - 4.18 |
| Spring DMY | KGD_GBLUP | 0.28 | 2.06 | -0.13 – 6.39 |
| GS | KGD_GBLUP | 0.25 | 3.20 | -0.76 - 20.4 |
| LS | KGD_GBLUP | 0.44 | 1.68 | 0.36 - 3.54 |
| Spring DMY | RKHS | 0.31 | 1.61 | 0.02 - 3.29 |
| GS | RKHS | 0.21 | 1.15 | -0.62 - 2.75 |
| LS | RKHS | 0.39 | 1.39 | 0.31 - 3.64 |

**Table S3:** Effect of training set size on the predictive ability (PA), bias and bias ranges of three traits DMY (dry matter) yield, GS (growth score) and LS (leaf size) combined across sites and years. Modelling implemented using GBLUP.

| **Trait** | **TS%** | **TS** | **PA** | **Bias** |
| --- | --- | --- | --- | --- |
| Spring DMY | 10% | 20 | 0.15 | 4.29 |
| Spring DMY | 20% | 40 | 0.12 | 5.77 |
| Spring DMY | 30% | 60 | 0.14 | 3.11 |
| Spring DMY | 40% | 80 | 0.19 | 65.12 |
| Spring DMY | 50% | 100 | 0.23 | 4.03 |
| Spring DMY | 60% | 120 | 0.24 | 3.48 |
| Spring DMY | 70% | 140 | 0.27 | 4.09 |
| Spring DMY | 80% | 160 | 0.27 | 5.15 |
| Spring DMY | 90% | 180 | 0.29 | 2.49 |
| Spring DMY | 100% | 200 | 0.29 | 2.17 |
| GS | 10% | 20 | -0.09 | 0.67 |
| GS | 20% | 40 | 0.11 | 2.60 |
| GS | 30% | 60 | 0.08 | 3.34 |
| GS | 40% | 80 | 0.10 | 2.08 |
| GS | 50% | 100 | 0.11 | 5.13 |
| GS | 60% | 120 | 0.14 | 3.00 |
| GS | 70% | 140 | 0.16 | 4.87 |
| GS | 80% | 160 | 0.18 | 3.97 |
| GS | 90% | 180 | 0.20 | 2.80 |
| GS | 100% | 200 | 0.21 | 2.36 |
| LS | 10% | 20 | 0.10 | 1.74 |
| LS | 20% | 40 | 0.21 | 6.07 |
| LS | 30% | 60 | 0.25 | 2.86 |
| LS | 40% | 80 | 0.30 | 2.43 |
| LS | 50% | 100 | 0.36 | 3.03 |
| LS | 60% | 120 | 0.35 | 2.30 |
| LS | 70% | 140 | 0.39 | 1.99 |
| LS | 80% | 160 | 0.39 | 1.75 |
| LS | 90% | 180 | 0.39 | 1.56 |
| LS | 100% | 200 | 0.42 | 1.59 |

**Table S4:** Effect of number of markers on the predictive ability (PA), bias and bias range of three traits DMY (dry matter) yield, GS (growth score) and LS (leaf size) combined across sites and years. Modelling implemented using GBLUP.

| **Trait** | **Markers%** | **Markers** | **PA** | **Bias** |
| --- | --- | --- | --- | --- |
| Spring DMY | 0.05% | 55 | 0.08 | 1.57 |
| Spring DMY | 0.10% | 110 | 0.13 | 1.38 |
| Spring DMY | 0.50% | 550 | 0.19 | 2.02 |
| Spring DMY | 1% | 1100 | 0.23 | 3.95 |
| Spring DMY | 5% | 5500 | 0.28 | 2.51 |
| Spring DMY | 10% | 11000 | 0.28 | 2.24 |
| Spring DMY | 50% | 55000 | 0.28 | 2.05 |
| Spring DMY | 100% | 110000 | 0.31 | 2.23 |
| GS | 0.05% | 55 | 0.06 | 0.87 |
| GS | 0.10% | 110 | 0.10 | 2.94 |
| GS | 0.50% | 550 | 0.13 | 4.37 |
| GS | 1% | 1100 | 0.16 | 2.18 |
| GS | 5% | 5500 | 0.20 | 2.38 |
| GS | 10% | 11000 | 0.20 | 3.23 |
| GS | 50% | 55000 | 0.20 | 2.36 |
| GS | 100% | 110000 | 0.21 | 2.36 |
| LS | 0.05% | 55 | 0.12 | 0.97 |
| LS | 0.10% | 110 | 0.16 | 2.89 |
| LS | 0.50% | 550 | 0.27 | 1.34 |
| LS | 1% | 1100 | 0.31 | 1.25 |
| LS | 5% | 5500 | 0.41 | 1.57 |
| LS | 10% | 11000 | 0.41 | 1.52 |
| LS | 50% | 55000 | 0.42 | 1.57 |
| LS | 100% | 110000 | 0.42 | 1.59 |

**Table S5:** Multi-trait predictive ability (PA), bias and bias range for primary traits: DMY (dry matter), combined across sites and years, SNPRS (pre-summer stolon number) and SNPOS (post-summer stolon number) using GS (growth score), LS (leaf size) SBPRS (pre-summer stolon branching) and SBPOS (post-summer stolon branching as secondary traits yield.

| **Trait** | **CV** | **PA** | **Average bias** |
| --- | --- | --- | --- |
| Spring DMY | Single | 0.29 | 0.04 |
| Spring DMY-GS | MTCV1 | 0.27 | 1.26 |
| Spring DMY-GS | MTCV2 | 0.65 | 1.97 |
| Spring DMY-GS+LS | MTCV1 | 0.29 | 1.36 |
| Spring DMY-GS+LS | MTCV2 | 0.66 | 1.98 |
| Spring DMY-LS | MTCV1 | 0.29 | 1.20 |
| Spring DMY-LS | MTCV2 | 0.37 | 1.44 |
| SNPRS | Single | 0.15 | 0.98 |
| SNPRS-SBPRS | MTCV1 | 0.15 | 0.75 |
| SNPRS-SBPRS | MTCV2 | 0.54 | 1.73 |
| SNPOS | Single | -0.11 | 1.14 |
| SNPOS-SBPOS | MTCV1 | -0.10 | -0.65 |
| SNPOS-SBPOS | MTCV2 | 0.28 | 1.56 |
